# A circuitous route for in vitro multi-enzyme cascade production of cytidine triphosphate to overcome the thermodynamic bottleneck

**DOI:** 10.1186/s40643-023-00724-6

**Published:** 2024-01-04

**Authors:** Zonglin Li, Yahui Zhong, Zhoulei Qing, Zhimin Li

**Affiliations:** 1 State Key Laboratory of Bioreactor Engineering, East China University of Science and Technology, 130 Meilong Road, Shanghai, 200237 China; 2 Shanghai Collaborative Innovation Center for Biomanufacturing Technology, 130 Meilong Road, Shanghai, 200237 China

**Keywords:** CTP, In vitro, Multi-enzymatic cascade, Circuitous route, Directed evolution, CTP synthetase

## Abstract

Cytidine triphosphate (CTP), as a substance involved in the metabolism of phospholipids, proteins and nucleic acids, has precise drug effects and is a direct precursor for the synthesis of drugs such as citicoline. In this study, we established an in vitro six-enzyme cascade system to generate CTP. To avoid thermodynamic bottlenecks, we employed a circuitous and two-stage reaction strategy. Using cytidine as the key substrate, the final product CTP is obtained via the deamination and uridine phosphorylation pathways, relying on the irreversible reaction of cytidine triphosphate synthase to catalyze the amination of uridine triphosphate. Several extremophilic microbial-derived deaminases were screened and characterized, and a suitable cytidine deaminase was selected to match the first-stage reaction conditions. In addition, directed evolution modification of the rate-limiting enzyme CTP synthetase in the pathway yielded a variant that successfully relieved the product feedback inhibition, along with a 1.7-fold increase in activity over the wild type. After optimizing the reaction conditions, we finally carried out the catalytic reaction at an initial cytidine concentration of 20 mM, and the yield of CTP exceeded 82% within 10.0 h.

**Graphical Abstract:**

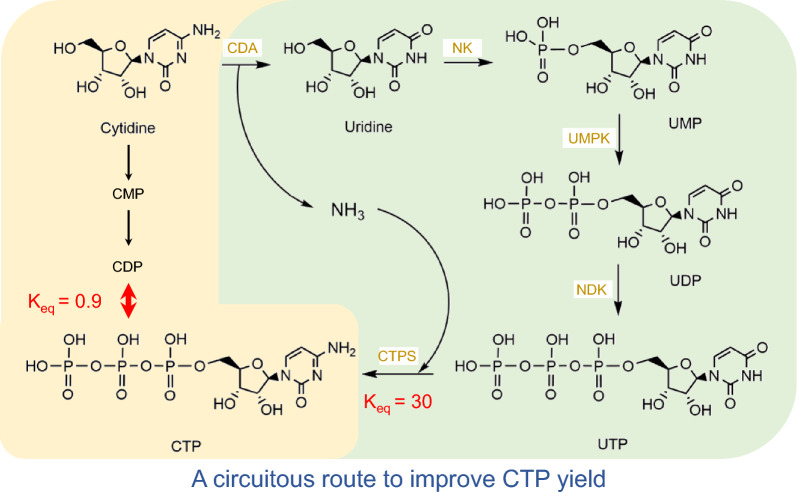

**Supplementary Information:**

The online version contains supplementary material available at 10.1186/s40643-023-00724-6.

## Introduction

The pyrimidine metabolic pathway has long been of interest to bioengineers, focusing on the production of a wide range of high-value-added chemicals in the pathway, including orotate (Carvalho et al. 2016), nucleotides, and their derivatives (Tang et al. 2010; Ren et al. 2021), which have a wide range of uses in food, nutraceuticals, and pharmaceuticals. For example, uridine is an important precursor for the synthesis of 5-trifluoromethyluridine and deoxyribonucleosides (Ghosh et al. 2019; Li et al. 2016). Cytidine triphosphate (CTP) has the function of regulating and promoting the repair of damaged neural tissues, improves fat metabolism, and also serves as an intermediate for the synthesis of drugs such as cytidine diphosphocholine (CDP-choline) and CDP-diacylglycerol (Marijani and Abonyo 2011; Jennings and Epand 2020). However, the pyrimidine metabolic pathway is extremely tightly regulated, e.g., uridine triphosphate (UTP) inhibits uridine monophosphate kinase (UMPK) activity; orotate inhibits dihydroorotate dehydrogenase activity (Fresquet et al. 2000; Lipscomb and Kantrowitz 2012). In addition, the lack of an efflux system results in a very limited range of nucleoside products that can be produced by fermentation (Qian et al. 2014). Therefore, in vitro enzymatic production of high-value-added substances in the pyrimidine metabolic pathway has attracted attention.

Natural enzymes are often not directly competent for in vitro catalytic tasks, and more often need to be engineered and modified, which includes improving catalytic activity, alleviating product or substrate inhibition, and changing substrate preference (Nirantar 2021). With the rapid development of synthetic biology, protein engineering approaches have received widespread attention. The earliest strategy was rational design based on an in-depth understanding of structural features and contribution to function (Osuna 2020). Directed evolution, which emerged at the turn of the century, is a powerful tool for modifying enzyme activity and has become the most widely used method (Bunzel et al. 2021). Directed evolution uses natural evolutionary strategies to create diverse libraries of mutants, and then identifies improved progeny from the libraries using appropriate selection methods. Besides, semi-rational design methods that target saturating mutations to multiple specific residues in the presence of functional or structural information are also very useful (Chica et al. 2005).

Although enzymes can reduce the activation energy of chemical reactions, neither wild enzymes nor variants obtained through complex engineering can change the equilibrium constant (*K*_eq_) of the reaction. It is well-known that the value of the *K*_eq_ is closely related to the thermodynamic function, and the two are linked through the Gibbs free energy (Δ*G*). When Δ*G* is negative, *K*_eq_ is greater than 1, implying that the reaction can proceed spontaneously. Conversely, when Δ*G* is positive, *K*_eq_ is less than 1, implying that the reaction tends to transform reactants rather than generate products. The thermodynamics of chemical reactions is essential to consider when designing a multi-enzyme cascade system. When a reversible or thermodynamically unfavorable reaction exists in the system, it generally prevents a high yield of the target product (Sun et al. 2021; Wang et al. 2017).

The conventional enzyme-catalyzed preparation of CTP was obtained by a multi-step enzyme-catalyzed reaction using cytidine or cytidine monophosphate (CMP) as substrate (Wen et al. 2023; Lee and Kim 2006). However, the *K*_eq_ value for the conversion of CDP to CTP is only 0.9, resulting in often poor yields of CTP. More seriously, this limitation is difficult to be broken through protein engineering methods. In this paper, we designed a circuitous route for the in vitro production of CTP by a six-enzyme cascade to overcome the thermodynamic bottleneck. The enzymes used include cytidine deaminase (CDA), nucleoside kinase (NK), UMPK, nucleoside diphosphate kinase (NDK), CTP synthase (CTPS), and polyphosphate kinase (PPK) for ATP regeneration. Cytidine was used as the starting substrate since cytidine is almost half the price of uridine and the ammonia produced by the reaction can be utilized by CTPS. CTPS catalyzes the irreversible ATP-dependent amination of UTP to form CTP, which has a Δ*G* of − 8.5 kJ/mol (at pH 8.0), suggesting that it is prone to proceed spontaneously. However, for CTPS, its ability to bind the substrate UTP and the product CTP is nearly identical, and thus it is strongly inhibited by the product CTP (Endrizzi et al. 2005). We obtained a variant with reduced feedback inhibition and improved activity through directed evolution. After optimizing the reaction conditions, 16.5 mM CTP was accumulated in 10.0 h with a yield of more than 82%.

## Materials and methods

### Strains, plasmids, chemicals, and culture media.

*Escherichia coli* TOP10 (Sangon Biotech) was used for plasmid amplification and preservation. *E. coli* BL21 (DE3) (Novagen) and plasmid pET28a (+) (Novagen) were used for protein production. Plasmids pET28a-*rbsK*, encoding the NK variant A220L/H252A from *Phorcysia thermohydrogeniphila* (*Pt*NK^A220L/H252A^), pET28a-*pyrH*, encoding the UMPK variant R117A from *Sulfurovum lithotrophicum* (*Sl*UMPK^R117A^), pET28a-*ndk*, encoding the NDK from *Desulfurella amilsii* (*Da*NDK), and pET28a-*ppk*, encoding the PPK from *S. lithotrophicum* (*Sl*PPK) were available in our laboratory (Li et al. 2022). All chemicals were purchased from Titan (Shanghai, China). The polyphosphate used in this study was sodium hexametaphosphate (polyP_6_). Luria–Bertani (LB) medium, supplemented with 50 μg/mL kanamycin, was used for *E. coli* cell growth and recombinant protein production.

### Construction of expression system

To screen for CDA that could work efficiently under acidic conditions, the CDA from three extremophilic bacteria, *Sulfurovum.* sp. NBC37-1 (WP_011980871.1), *S. riftiae* (WP_082792074.1), and *Thermovibrio guaymasensis* (WP_121171554.1), which might meet our expectations, were selected for further study (Haridon et al. 2006; Giovannelli et al. 2016). The *cda* gene of *Sulfurovum.* sp. NBC37-1, *S. riftiae*, and *T. guaymasensis* were synthesized by Sangon Biotech (Shanghai, China) and introduced into the *Nco* I/*Xho* I restriction sites of pET28a (+). The CTPS used in this paper was derived from *Aquifex aeolicus* (WP_010880855.1). The *pyrG* gene of *A. aeolicus* was synthesized by Sangon Biotech (Shanghai, China) and introduced into the *Nco* I/*Xho* I restriction sites of pET28a (+). To enhance the gene expression level, DNA sequences were codon-optimized for *E. coli* codon usage. Then, the recombinant plasmids were transformed into *E. coli* BL21 (DE3) for CDAs and CTPS production.

### Expression and purification of enzymes

Expression and purification of the enzymes were as described previously (Li et al. 2022). The recombinant cells were incubated in 3 mL LB medium at 37 °C and 220 rpm for 6.0 h. Next, 2 mL of the cultures were transferred to 100 mL of newly prepared LB medium in 500-mL flasks and cultivated at 37 °C. The overexpression of the enzymes was induced by the addition of 0.2 mM of isopropyl-β-d-thiogalactopyranoside (IPTG) when the optical density at 600 nm of the culture reached 0.6, and then the bacteria were incubated at 18 °C and 220 rpm for 16.0 h. After induction, the cells were harvested by centrifugation at 4600×*g* and 4 °C for 5 min, washed once and resuspended in 20 mM Tris–HCl buffer (pH 8.0). The cells were then lysed by a high-pressure homogenizer supplied by the Shanghai Litu Mechanical Equipment Engineering Co., Ltd. (Shanghai, China). The lysate was centrifuged at 6000×*g* and 4 °C for 20 min. The protein in the supernatant was purified by Ni-chelating affinity chromatography. The purity of the enzyme was examined by SDS-PAGE (Additional file 1: Figure S1), and the proteins were quantified by the BCA Protein Assay Kit from Tiangen (Beijing, China).

### Directed evolution and high-throughput screening

Mutations were introduced by PCR. All mutants were constructed on the plasmid template encoding the wild-type protein. The primers used for mutation are listed in Additional file 1: Table S1, and all the open reading frames encoding mutants were sequenced by Sangon Biotech (Shanghai, China). The mutated plasmids were transformed into *E. coli* BL21 (DE3) for protein expression.

The method of establishing the saturation mutation library of *Aa*CTPS is as follows: colonies were randomly picked and inoculated into 96-well plates containing 100 μL LB medium per well with 50 μg/mL kanamycin. After incubation at 37 °C overnight, 6-μL cultures were transferred to 300 μL of fresh auto-inducing medium (10 g/L tryptone, 5 g/L yeast extract, 25 mM Na_2_HPO_4_, 25 mM KH_2_PO_4_, 50 mM NH_4_Cl, 5 mM Na_2_SO_4_, 2 mM MgSO_4_, 5 g/L glycerol, 0.5 g/L glucose, and 2 g/L α-lactose) (Studier 2005) with 50 μg/mL kanamycin in new 96 deep-well plates. Growth was allowed at 37 °C for 4 h, and then incubated at 18 °C for 16.0 h. 50 μL of cultures were harvested by centrifugation. The collected cells were lysed by freezing at − 80 °C twice and thawing.

High-throughput screening methods used to screen for *Aa*CTPS variants with relieved feedback inhibition were as follows: the standard assay mixture contained 100 mM Tris–HCl buffer (pH 7.0), 20 mM MgCl_2_, 10 mM ATP, 10 mM UTP, 10 mM CTP, and 10 mM glutamine. The collected cells were resuspended in the standard assay mixture to a final volume of 400 μL and then incubated at 45 °C for 5.0 h. Using ammonium molybdate spectrophotometric method to determine total phosphorus in reaction solution (Yang et al. 2007). More phosphorus could be detected in the reaction solution of the variant that relieves the feedback inhibition.

### Structure prediction and molecular docking

The structure of *Aa*CTPS and variants was predicted by the RoseTTAFold tool (https://github.com/RosettaCommons/RoseTTAFold). The optimized structures of different substrates were respectively docked with enzymes through Schrodinger 12.0 to investigate the molecular mechanism of substrate binding. The grid box was set to cover the substrate-binding pocket with a size of 6 Å and the center of the pocket was defined as the center of the box. Box and substrate with the optimal conformation were selected and the binding energy was calculated by the MM/GBSA module of the Schrodinger software. The precision of molecular docking was XP (extra precision).

### Effects of pH and temperature

The effect of pH on *Ss*CDA, *Sr*CDA, and *Tg*CDA was determined at 50 °C in buffers with different pH values from 4.0 to 8.5: phosphate buffer (PB buffer, pH 4.0–7.0), and Tris–HCl buffer (pH 7.0–8.5), respectively. The enzyme activity was measured directly at different pH conditions. The highest activity of the enzyme was set as 100% of relative activity. The effect of temperature on *Ss*CDA, *Sr*CDA, and *Tg*CDA was determined in PB buffer (pH 7.0). Measurements were performed at 25, 30, 37, 45, 50, 60, 70, 80, and 90 °C, respectively, and the highest activity of the enzyme under the various temperature conditions was set as 100% of relative activity.

### Enzyme activity and kinetic assays

The activity of *Sr*CDA was monitored by observing the generation of uridine via high-performance liquid chromatography (HPLC). The reaction was carried out in a 500-μL reaction mixture containing 10 mM cytidine in 50 mM PB buffer (pH 5.0) and an appropriate amount of enzyme. Routine assays were performed at 50 °C with a 3-min incubation time and immediately terminated by adding an equal volume of 1 M HCl on ice. One unit of activity was defined as the amount of enzyme that released 1 μmol of uridine per minute. Kinetic analyses were carried out toward uridine. All reactions were carried out at 50 °C in 50 mM PB buffer (pH 5.0). Samples were taken at 1, 2, 3, and 4 min of the reaction and kinetic parameters were calculated by measuring the uridine concentration. Substrate concentrations varied from 0.5 to 20 mM.

The activity of *Aa*CTPS and variants was monitored by observing the generation of CTP via HPLC. The reaction was carried out in a 500-μL reaction mixture containing 20 mM MgCl_2_, 10 mM ATP, 10 mM UTP, 10 mM glutamine in 50 mM PB buffer (pH 7.0), and an appropriate amount of enzyme. Routine assays were performed at 45 °C with a 5-min incubation time and immediately terminated by adding an equal volume of 1 M HCl on ice. One unit of activity was defined as the amount of enzyme that released 1 μmol of CTP per minute. Kinetic analyses were carried out toward UTP and ATP. All reactions were carried out at 45 °C in 50 mM PB buffer (pH 7.0). Samples were taken at 1, 2, 3, and 4 min of the reaction and kinetic parameters were calculated by measuring the CTP concentration. Substrate concentrations varied from 0.2 to 5.0 mM.

### Effect of initial CTP concentration

The inhibitory effect of CTP on the *Aa*CTPS and variants activity was tested, respectively. The reactions were conducted in 50 mM PB buffer (pH 7.0) at 45 °C containing 20 mM MgCl_2_, 10 mM ATP, 10 mM UTP, 10 mM glutamine, 0.1 g/L of the enzyme, and 5 mM CTP. Reactions were stopped after 5 min by adding an equal volume of 1 M HCl on ice. The samples were diluted and analyzed by measuring the concentration of CTP.

### Cascade reaction

For UMP synthesis, the reaction was conducted under the following conditions: 10 mL of 50 mM PB buffer (pH 5.0) containing 20 mM cytidine, 2 mM ATP, 5 mM polyP_6_, 20 mM MgCl_2_, 5.0 U/mL *Sr*CDA, and 30.0 U/mL *Sl*PPK at 50 °C (in a dry thermostatic metal bath reactor) for 1.0 h. When all of the cytidine was converted to uridine, 5.0 U/mL *Pt*NK^A220L/H252A^ was added to continue the reaction for 1.0 h under the same reaction conditions.

For CTP synthesis, before adding the remaining enzymes in the same pot, the pH of the UMP solution generated was adjusted to 8.0. Then, 20 mM polyP_6_, 40 mM MgCl_2_, 5.0 U/mL *Sl*UMPK^R117A^ variant, 5.0 U/mL *Da*NDK, and 10.0 U/mL *Aa*CTPS^I19T/R34G/R262H^ variant were added to produce CTP at 45 °C for 8.0 h (in a dry thermostatic metal bath reactor).

### HPLC analysis

The concentration of uridine and UMP was detected through HPLC (Shimadzu, Nexera XR) equipped with a C18 column (4.6 × 150 mm, InertSustain, Shimadzu-GL). The mobile phase was composed of 2.0 g/L KH_2_PO_4_ and 0.85 g/L K_2_HPO_4_: methanol (97:3, v/v) at a flow rate of 0.6 mL/min. The column temperature was maintained at 30 °C and the detection wavelength was set at 270 nm. Samples were first diluted in water. They were then filtered (0.22-μm hydrophilic PTFE syringe filter, ANPEL, Shanghai). Finally, 10 µL was applied to the column. The data were analyzed with Dionex Chromeleon software.

The concentration of uridine diphosphate (UDP), UTP, and CTP was detected through HPLC (Shimadzu, Nexera XR) equipped with a C18 column (4.6 × 150 mm, InertSustain, Shimadzu-GL). The mobile phase was composed 0.6% phosphoric acid-triethylamine (pH 6.5): methanol (97:3, v/v) at a flow rate of 0.6 mL/min. The column temperature was maintained at 30 °C and the detection wavelength was set at 270 nm. Samples were first diluted in water. They were then filtered (0.22-μm hydrophilic PTFE syringe filter, ANPEL, Shanghai). Finally, 10 µL was applied to the column. The data were analyzed with Dionex Chromeleon software (Additional file 1: Figure S2).

## Results and discussion

### Overview of the designed pathway

**Fig. 1:**
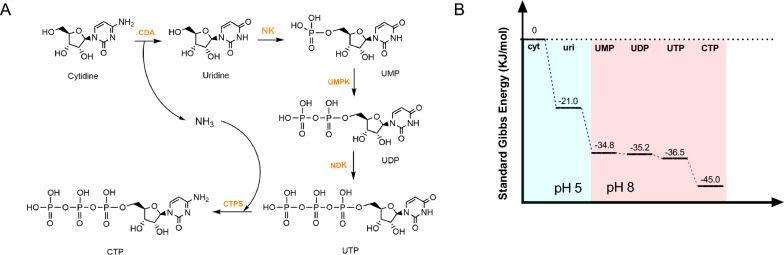
**A** Scheme of the in vitro multi-enzyme cascade for the synthesis of CTP. The enzymes are listed in Table 1. **B** The standard ΔG of the two-stage reaction. Data from http://equilibrator.weizmann.ac.il/

The conventional CTP enzymatic synthesis method starts from cytidine and undergoes a multi-step phosphorylation. However, after calculating the ΔG for each step of the reaction, it is not difficult to find that there is a thermodynamic bottleneck for the phosphorylation of CDP to CTP under both acidic and basic conditions (Additional file 1: Figure S3). To overcome the bottleneck, we designed the reaction route as shown in Fig. 1, and the details of the enzymes used are shown in Table 1. This route also uses cytidine as the initial substrate, but takes a small detour to utilize the phosphorylation pathway of uridine, and finally CTPS is responsible for catalyzing the generation of CTP. CTPS can catalyze the amination of UTP to give CTP using the amino group provided by glutamine, ammonia, or inorganic ammonium salts (Bearne et al. 2001). Under alkaline conditions, the ΔG of the CTPS-catalyzed reaction was − 8.5 kJ/mol, suggesting that the reaction occurs readily.

**Table 1:** Key information about the enzymes used in this study

Enzyme name	Microorganism source	Specific activity (U/mg)	Refs.
Cytidine deaminase	*Sulfurovum riftiae*	38.8 ± 0.4	This article
Nucleoside kinase^a^	*Phorcysia thermohydrogeniphila*	36.5 ± 0.7	Li et al. (2022)
UMP kinase^b^	*Sulfurovum lithotrophicum*	37.1 ± 1.1	Li et al. (2022)
Nucleoside diphosphate kinase	*Desulfurella amilsii*	450.9 ± 4.3	Li et al. (2022)
CTP synthase^c^	*Aquifex aeolicus*	2.7 ± 0.1	This article
Polyphosphate kinase	*Sulfurovum lithotrophicum*	180.5 ± 2.6	Sun et al. (2021)

^a^A220L/H252A variant of *Pt*NK

^b^R117A variant of *Sl*UMPK

^c^I19T/R34G/R262H variant of *Aa*CTPS

NKs can phosphorylate a variety of nucleosides to generate monophosphate nucleosides (Park and Gupta 2013). Previously, we modified the *P. thermohydrogeniphila*-derived NK to obtain a variant A220L/H252A with a tenfold increase in activity (Li et al. 2022). Considering that the activity of the *Pt*NK variant is almost completely lost in environments with pH greater than 5.5 and does not match the CTPS-catalyzed reaction conditions, we divided the coupling reaction into two stages. For convenience, in the following, we use stage I and stage II to represent the generation of UMP and CTP, respectively. Often, it is important to choose the right buffer for the in vitro reaction. Since the two-stage reaction needs to be carried out at different pH values, we directly used PB buffer. CTP was synthesized in a one-pot, two-stage process using 20 mM cytidine as the substrate. The pH of the solution was adjusted after 2.0 h of reaction in the first stage, and then the enzyme required for the second stage was added and the reaction was continued for 8.0 h.

### Screening and characterization of CDAs

Previously, we utilized the *Pt*NK coupled with PPK for low-cost in vitro synthesis of nucleotides (Li et al. 2021). The coupling system operates at a pH of 5.0 and an optimal reaction temperature of 50 °C. Therefore, the optimal working conditions for CDA, which catalyzes the previous reaction to generate uridine, should ideally be the same. To meet our needs, we screened three CDAs of extremophilic origin, which is because extremophilic enzymes tend to have special properties, such as stronger tolerance to extreme conditions (acidic or alkaline environments, high temperatures, etc.) (Atalah et al. 2019; Sammond et al. 2016).

**Fig. 2:**
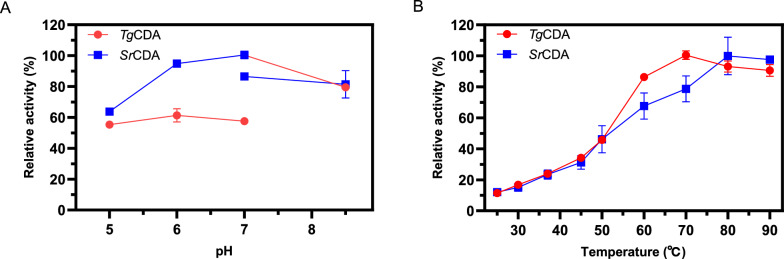
Effect of pH (**A**) and temperature (**B**) on CDAs. The effect of pH was determined at 50 °C in buffers with different pH values. The effect of temperature was determined in PB buffer (pH 7.0) at pH 5.0. The reaction system consisted of 10 mM cytidine, 5 μmol of various pure enzymes. All measurements were carried out in triplicates

To investigate the optimum pH of *Ss*CDA, *Sr*CDA, and *Tg*CDA, the enzyme activity was analyzed under different pH values. Figure 2A shows the pH profile of the enzyme activity at 50 °C. Except for *Ss*CDA, which did not exhibit deaminase activity under any of the conditions tested, the remaining two enzymes had maximum activity at pH 7.0. Unlike *Sr*CDA, which performed better in PB buffer, *Tg*CDA did the opposite, preferring Tris–HCl buffer. Notably, both enzymes maintained at least 60% of their highest activity at pH 5.0, indicating a match with the first-stage reaction conditions. The effect of temperature on the enzyme activities of *Ss*CDA, *Sr*CDA, and *Tg*CDA was also investigated. Figure 2B displays a clear relationship between the temperature and the enzyme activity. Similarly, *Ss*CDA was unable to catalyze the cytidine deamination reaction under any conditions, indicating that the substrate of *Ss*CDA does not include cytidine. *Sr*CDA and *Tg*CDA activity increased with increasing temperature from 25 to 80 °C, which is consistent with the nature of thermophilic enzymes. In addition, after calculation we found that the specific activity of *Sr*CDA was at least 10 times higher than that of *Tg*CDA in all cases tested (Additional file 1: Table S2). We further tested the activity and kinetic parameters of *Sr*CDA at pH 5.0 and 50 °C. Kinetic rate measurements of the substrate revealed that *Sr*CDA was obeying standard Michaelis–Menten behavior, with Km values of 2.5 ± 0.1 mM toward cytidine, Vmax value of 388.4 ± 8.5 μmol min^−1^. The catalytic efficiency (*k*_cat_/Km) of the *Sr*CDA toward cytidine was 4.7 ± 0.1 s^−1^ mM^−1^. We chose *Sr*CDA for subsequent experiments.

Zn^2+^ is very common in deaminase-catalyzed reactions as an important cofactor (Xiang et al. 1996). To optimize Zn^2+^ concentration, we respectively added 0, 0.5, 1, 5, and 10 mM Zn^2+^ into the system while maintaining other reaction conditions constant. The optimal Zn^2+^ concentration was determined by calculating the rate of uridine production over 10 min. Unexpectedly, we found that *Sr*CDA did not require Zn^2+^ to assist catalysis. Instead, as more Zn^2+^ was added, the rate of uridine production was slower. A previous study of the optimal cofactor concentration for cytidine deaminase found that the enzyme was active even without the addition of Zn^2+^. This is due to the fact that Zn^2+^ present in the medium components binds strongly to the protein and cannot be easily eluted (Carlow et al. 1999). For *Sr*CDA, the situation may be very similar. A small amount of Zn^2+^ in the medium is sufficient for the enzyme to function properly.

Finally, the first stage of UMP production is carried out according to the optimal conditions. Since the substrate of NKs also includes cytidine, after all the cytidine in the reaction system was converted to uridine, 5.0 U/mL *Pt*NK^A220L/H252A^ was added and the reaction was continued for 1.0 h under the same reaction conditions. Finally, 20 mM cytidine was converted to 19.8 mM UMP for the second stage of CTP preparation.

### Results of directed evolution of AaCTPS

Like all other CTPSs, *Aa*CTPS catalyzes ATP-dependent amination of UTP to CTP. According to the results of alphafold modeling, the structural domain formed by amino acids 1–265 at the N-terminal of *Aa*CTPS is responsible for catalyzing the formation of CTP, and we therefore hypothesized that the product CTP-binding site should be at the N-terminal of the protein. Random mutations were introduced into the N-terminus of *Aa*CTPS using error-prone PCR to create a mutation library containing 28,800 samples. After the high-throughput screening, 24 candidate variants were obtained for re-screening validation. The crude enzyme assay showed that only one variant relieved the inhibition of CTP. The results of the pure enzyme assay showed that in the initial reaction system containing 5 mM CTP, the variant produced 9.6 mM CTP after 4.0 h of catalysis, and the UTP was basically consumed, whereas the wild type accumulated only 2.1 mM CTP, which indicated that the variant alleviated the feedback inhibition. Sequencing results showed that the variant was I19T/R34G/R262H.

To understand the specific effects of the three mutation sites on the enzyme, we further investigated six reduction mutations (including three single and three double mutations) in the I19T/R34G/R262H variant. All variants were first tested for specific activity and the results are shown in Table 2. As can be seen from the data in the table, the I19T mutation contributed the most to the increase in activity, and the specific activity of the I19T/R34G/R262H variant was 1.7 times higher than that of the wild enzyme. Finally, all variants were tested for resistance to CTP inhibition. Wild enzyme activity was inhibited by more than 90% when 5 mM CTP was initially present in the system. Three variants, I19T, I19T/R34G, and I19T/R262H, retained more than 80% of the highest value. The triple mutation goes even further, with activity virtually unaffected.

**Table 2:** The specific activity of *Aa*CTPS and the seven variants

Enzyme	Specific activity (U/mg)
WT	1.55 ± 0.04
I19T/R34G/R262H	2.68 ± 0.07
I19T	2.32 ± 0.09
R34G	1.73 ± 0.01
R262H	1.56 ± 0.02
I19T/R34G	1.20 ± 0.03
I19T/R262H	2.07 ± 0.10
R34G/R262H	1.93 ± 0.06

### In-depth analysis of the mechanisms

**Fig. 3:**
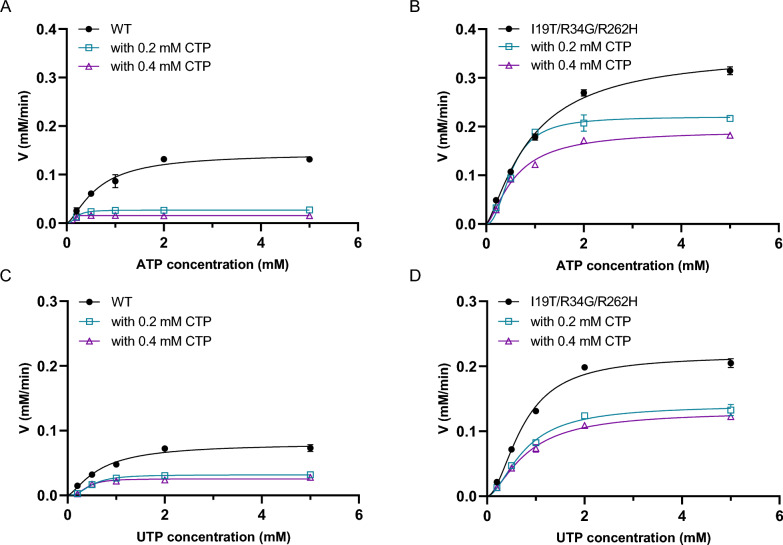
Kinetic results of wild-type (**A**) and the variant (**B**) on ATP in the presence of different concentrations of CTP. Kinetic results of wild-type (**C**) and the variant (**D**) on UTP in the presence of different concentrations of CTP. Fitting was performed using GraphPad software, and the allosteric sigmoidal model was selected. All measurements were carried out in triplicates

The kinetic constants of the wild enzyme and the I19T/R34G/R262H variant for the substrates ATP and UTP were tested in the absence and with the addition of either 0.2 mM or 0.4 mM CTP (Fig. 3 and Table 3). The affinity of the variant for UTP was significantly lower than that of the wild-type enzyme. This is as expected because the binding site for CTP is almost identical to that of UTP, and relieving the CTP feedback inhibition means that the common CTP/UTP binding site may be disrupted.

**Table 3:** Kinetic data of wild type and the I19T/R34G/R262H variant on ATP and UTP when different concentrations of CTP are present

Enzyme	Sub	*V*max (μM/min)			*K*_M_ (mM)			*k*_cat_ (s^−1^)		
	CTP (mM)	0	0.2	0.4	0	0.2	0.4	0	0.2	0.4
WT	ATP^a^	131.0 ± 2.0	26.9 ± 2.2	23.0 ± 3.0	1.08 ± 0.05	0.44 ± 0.02	–^c^	1.31 ± 0.01	0.26 ± 0.03	0.23 ± 0.01
variant		314.8 ± 6.2	208.6 ± 1.8	177.4 ± 2.4	0.77 ± 0.06	0.61 ± 0.01	0.48 ± 0.02	3.14 ± 0.05	2.08 ± 0.07	1.77 ± 0.02
WT	UTP^b^	80.2 ± 3.5	31.8 ± 1.3	25.5 ± 2.3	0.53 ± 0.01	0.17 ± 0.03	0.13 ± 0.05	0.80 ± 0.01	0.32 ± 0.02	0.25 ± 0.02
variant		210.9 ± 5.2	133.5 ± 3.2	124.3 ± 4.7	0.79 ± 0.06	0.82 ± 0.01	0.94 ± 0.05	2.10 ± 0.09	1.33 ± 0.02	1.24 ± 0.01

^a^ATP concentrations range from 0.2 to 5.0 mM and UTP concentrations are fixed at 10 mM

^b^UTP concentrations range from 0.2 to 5.0 mM and ATP concentrations are fixed at 10 mM

^c^Not detected

**Fig. 4:**
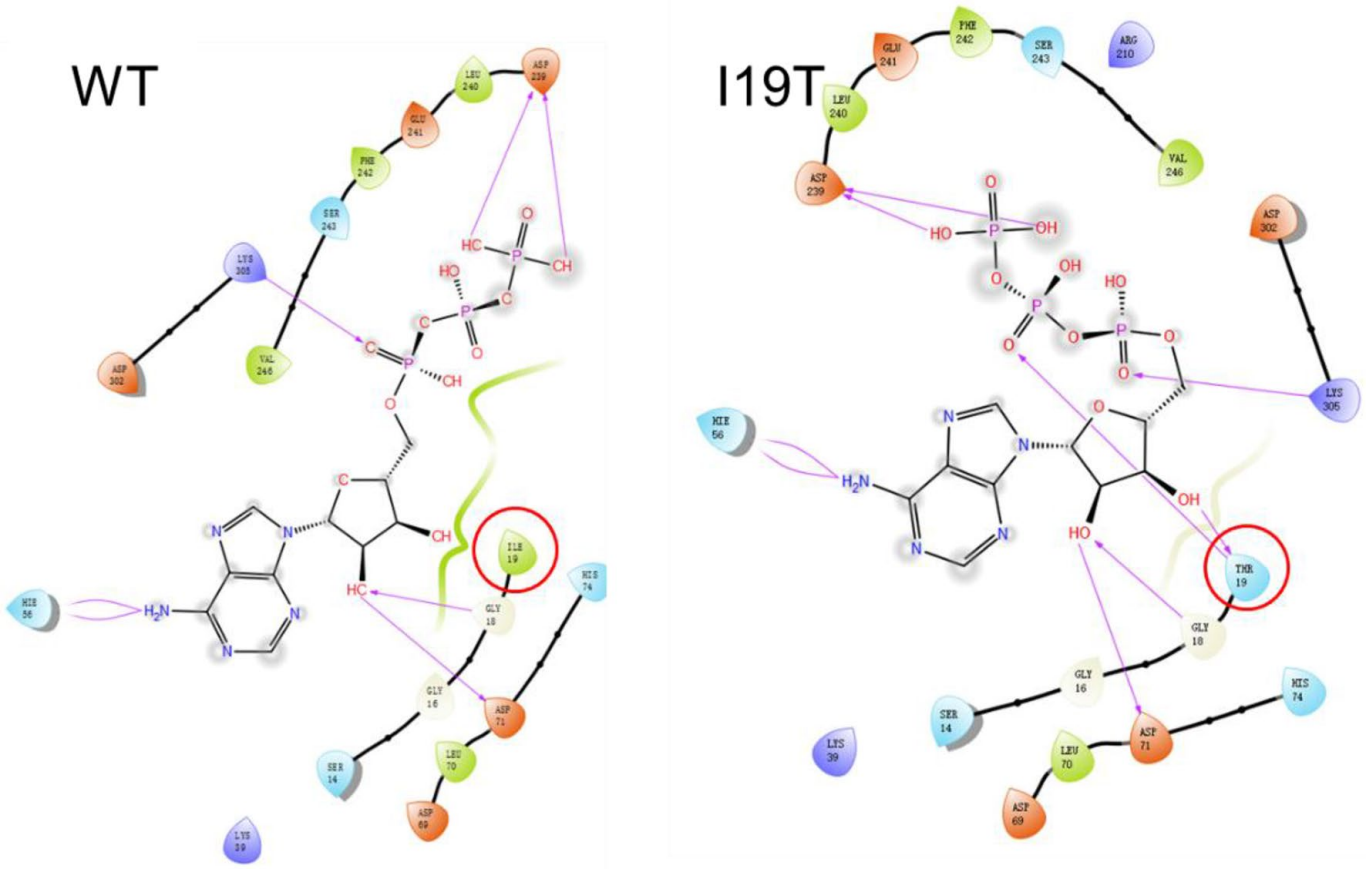
Effect of the I19T mutation on ATP binding. Replacement of the isoleucine at position 19 by a threonine significantly enhanced the binding of the enzyme to ATP

The models of *Aa*CTPS and the I19T/R34G/R262H variant were predicted using RoseTTAFold online website and molecular docking was performed by Schrodinger software, and the results are shown in 2D plots. The replacement of the I19 site by a threonine added a new hydrogen bonding interaction between the enzyme and the substrate ATP (Fig. 4). The binding energies of the I19T/R34G/R262H variant and wild enzyme to ATP were calculated by MM/GBSA to be − 53.71 and − 38.44 kJ/mol, respectively, indicating the contribution of the I19T mutation to ATP binding.

Combining structural and kinetic data, we conclude that although the wild enzyme binds UTP more readily, this means that it is also more susceptible to CTP inhibition. Besides, because the wild enzyme has a lower affinity and number of conversions for ATP, it is the utilization of ATP that is the major limiting factor for the apparent activity of the enzyme. The relieving of CTP inhibition by the I19T/R34G/R262H variant means that the binding sites for CTP and UTP may be disrupted, as evidenced by a decrease in affinity for UTP and conversion number decreased, but the specificity constant for ATP was increased, and instead of being the limiting factor, the utilization of UTP reached a near 1:1 equilibrium with the rate of ATP utilization, resulting in an increase in apparent activity compared to the wild enzyme.

### In vitro six-enzyme cascade synthesis of CTP

**Fig. 5:**
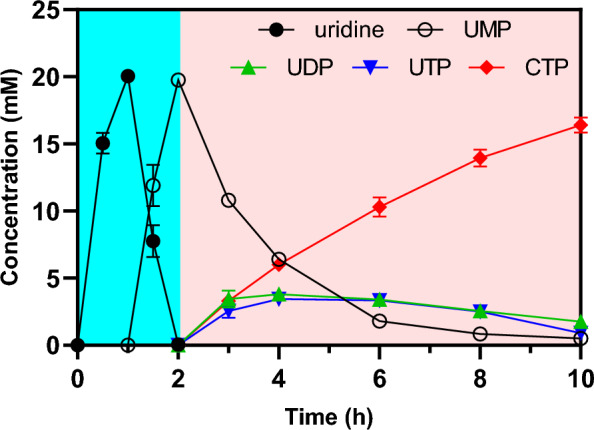
Two-stage synthesis of CTP using 20 mM cytidine as substrate. The reaction was initiated at 50 °C in 50 mM PB buffer (pH 5.0) containing 2 mM ATP, 5 mM polyP_6_, 20 mM MgCl_2_, and pure enzyme to generate UMP. After 2.0 h of the first-stage reaction, the pH and temperature of the solution were adjusted, and the remaining enzyme and substrate were added to produce CTP. All measurements were carried out in triplicates

The CTP production reaction was carried out according to the system described in methods using the screened *Sr*CDA and the modified *Aa*CTPS^I19T/R34G/R262H^. Since the optimum pH of *Sl*UMPK, *Da*NDK, and *Aa*CTPS were all 8.0, no optimization of the pH of the second stage was required. Considering that the half-life of the *Sl*UMPK^R117A^ variant at 50 °C was also not very excellent to ensure a long-term reaction, 45 °C was chosen as the reaction temperature for the second stage. According to the previous judgment, the rapid consumption of UTP was necessary because it would break the equilibrium of the NDK-catalyzed reversible reaction. Therefore, it was decided to use 10.0 U/mL *Aa*CTPS^I19T/R34G/R262H^. After obtaining 19.8 mM UMP in the first stage, the pH of the solution containing UMP was adjusted and the enzyme required for the second stage was added. After 8.0 h of reaction, 16.5 mM CTP was detected, with the remaining 0.5 mM UMP, 1.8 mM UDP, and 0.9 mM UTP (Fig. 5). The above results indicate that our established coupling reaction successfully avoided the thermodynamic bottleneck and improved the yield of CTP.

## Conclusions

In this paper, we constructed a circuitous route for in vitro six-enzyme cascade production of CTP to break through the thermodynamic bottleneck. An extremophilic microbial-derived cytidine deaminase, *Sr*CDA, was obtained by screening, which could work under operating conditions of pH 5.0 and 50 °C, and was successfully matched with other reactions in the first stage. The key rate-limiting enzyme *Aa*CTPS was also modified by directed evolution to obtain the I19T/R34G/R262H variant with increased activity while relieving feedback inhibition. Further detailed determination of the kinetic parameters of the variant and the six reduced mutants, combined with structural analysis, determined that the substitution of threonine for isoleucine at position 19 increased the hydrogen bonding force with the substrate ATP. Finally, the in vitro production of 16.5 mM CTP was realized, with a conversion rate of more than 82% to the substrate cytidine, laying the foundation for the production of more nucleotide derivatives.

### Supplementary Information

**Additional file 1: Figure S1.** SDS-PAGE analysis of three CDAs. **Figure S2.** HPLC data. **Figure S3.** ΔG data. **Table S1.** Primer sequences. **Table S2.** Specific activities of *Sr*CDA and *Tg*CDA.

## Data Availability

All data generated or analyzed during this study are included in this article and Additional file 1.

## Abbreviations

ATP: Adenosine triphosphate
CTP: Cytidine triphosphate
UMP: Uridine monophosphate
UDP: Uridine diphosphate
UTP: Uridine triphosphate
Keq: Equilibrium constant
ΔG: Gibbs free energy
polyP_6_: Hexametaphosphate
PPK: Polyphosphate kinase
CDA: Cytidine deaminase
NK: Nucleoside kinase
UMPK: UMP kinase
NDK: Nucleoside diphosphate kinase
CTPS: CTP synthase
PB buffer: Phosphate buffer
Tris–HCl buffer: Tris(hydroxymethyl)aminomethane buffer
LB medium: Luria–Bertani medium
SDS-PAGE: Sodium dodecyl sulfate–polyacrylamide gel electrophoresis
PCR: Polymerase chain reaction
IPTG: Isopropyl β-d-1-thiogalactopyranoside
HPLC: High-performance liquid chromatography
