# Supplementary material for: A circuitous route for in vitro multi-enzyme cascade production of cytidine triphosphate to overcome the thermodynamic bottleneck
Source: Bioresour Bioprocess. 2024 Jan 4;11(1):6. doi: 10.1186/s40643-023-00724-6 (PMC10992187; doi:10.1186/s40643-023-00724-6)
Supplement: Supplementary file 1 — Additional file 1: Figure S1. SDS-PAGE analysis of three CDAs. Figure S2. HPLC data. Figure S3. ΔG data. Table S1. Primer sequences. Table S2. Specific activities of SrCDA and TgCDA. [file 40643_2023_724_MOESM1_ESM.docx]

**Additional Information**

**A circuitous route for *in vitro* multi-enzyme cascade production of cytidine triphosphate to overcome the thermodynamic bottleneck**

Zonglin Li^a#,†^, Yahui Zhong^a,†^, Zhoulei Qing^a^, Zhimin Li^a,b#^

^a^ State Key Laboratory of Bioreactor Engineering, East China University of Science and Technology, 130 Meilong Road, Shanghai 200237, China

^b^ Shanghai Collaborative Innovation Center for Biomanufacturing Technology, 130 Meilong Road, Shanghai 200237, China

^†^ These authors contributed equally.

**^#^Corresponding author:**

Zonglin Li

**E-Mail:** lzlinn@ecust.edu.cn

Zhimin Li

**E-Mail:** [lizm@ecust.edu.cn](mailto:lizm@ecust.edu.cn)

**Table S1. Primers used for mutation.**

| F1 | CGAACACCTGAACCTGGAACACCGCGAAGTGAACCTGGGCAAATG |
| --- | --- |
| R1 | CAGTGGTGGTGGTGGTGGTGCTCGAGGGTGAATTTACGTTTGTAT |
| DR-F1 | TTAACTTTAAGAAGGAGATATACCATGGGCATGGCGAAATACATC |
| DR-R1 | GCGGTGTTCCAGGTTCAGGTGTTCGGTGATAATACGATCGATACC |

Error-prone PCR using the pair of primers DR-F1 and DR-R1, and conventional PCR using the pair of primers F1 and R1, using the wild *Aa*CTPS plasmid as the template.

**Table S2. Specific activities of *Sr*CDA and *Tg*CDA at different conditions.**

| *Sr*CDA  (U/mg) | 25°C | 30°C | 37°C | 45°C | 50°C | 60°C | 70°C | 80°C | 90°C |
| --- | --- | --- | --- | --- | --- | --- | --- | --- | --- |
| pH 5 | 9.5^a^ | 11.0 | 18.2 | 22.6 | 38.8 | 56.6 | 66.4 | 76.5 | 73.2 |
| pH 6 |  |  |  |  | 59.2 |  |  |  |  |
| pH 7 |  |  |  |  | 62.5 |  |  |  |  |
| pH 8.5 |  |  |  |  | 47.4 |  |  |  |  |

| *Tg*CDA  (U/mg) | 25°C | 30°C | 37°C | 45°C | 50°C | 60°C | 70°C | 80°C | 90°C |
| --- | --- | --- | --- | --- | --- | --- | --- | --- | --- |
| pH 5 | 0.7 | 0.7 | 0.9 | 1.4 | 2.1 | 3.3 | 4.2 | 3.9 | 3.3 |
| pH 6 |  |  |  |  | 2.2 |  |  |  |  |
| pH 7 |  |  |  |  | 4.0 |  |  |  |  |
| pH 8.5 |  |  |  |  | 3.1 |  |  |  |  |

^a^ One unit of activity was defined as the amount of enzyme that released 1 μmol of uridine per minute.

**Supplementary Figure captions:**

Figure S1: SDS-PAGE analysis of three CDAs. M: protein marker; 1: *Tg*CDA; 2: *Sr*CDA; 3: *Ss*CDA.

Figure S2: The composition of the reaction solution was analyzed by HPLC. CTP exhibited a retention time of approximately 5.5 min, UMP was near 6.8 min, UDP was near 9.2 min, while UTP was near 13.8 min.

Figure S3: The standard ΔG of the conventional enzyme-catalyzed preparation of CTP shows a thermodynamic bottleneck for the phosphorylation of CDP to CTP under both acidic and basic conditions. Data from <http://equilibrator.weizmann.ac.il/>.

**Figure S1**

**
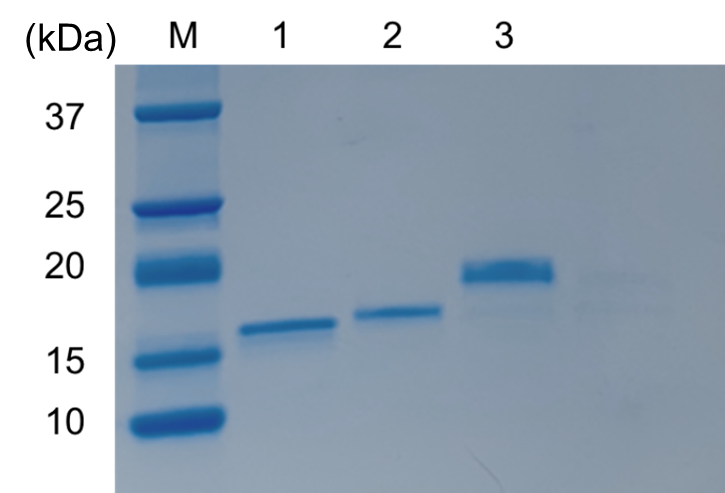
**

**Figure S2**


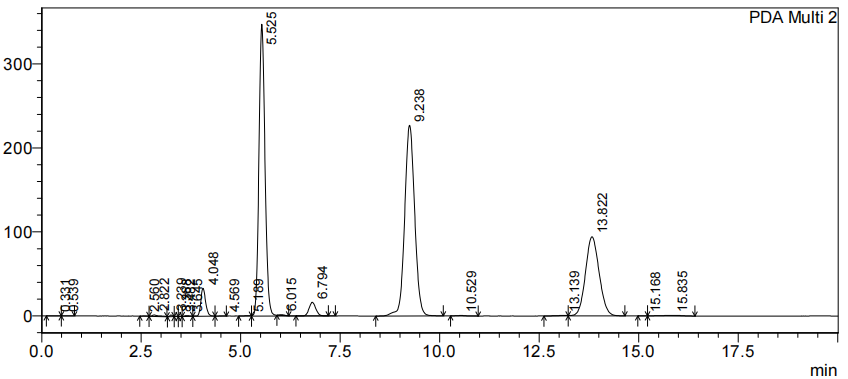


**Figure S3**
